# Experimental Study on Plasmodium berghei, Anopheles Stephensi, and BALB/c Mouse System: Implications for Malaria Transmission Blocking Assays

**Published:** 2018

**Authors:** Hossein DEHGHAN, Mohammad Ali OSHAGHI, Seyed Hassan MOSA-KAZEMI, Mohammad Reza ABAI, Fatemeh RAFIE, Mehdi NATEGHPOUR, Habib MOHAMMADZADEH, Leila FARIVAR, Mulood MOHAMMADI BAVANI

**Affiliations:** 1. Dept. of Medical Entomology & Vector Control, School of Public Health, Tehran University of Medical Sciences, Tehran, Iran; 2. Dept. of Parasitology and Medical Mycology, School of Public Health, Tehran University of Medical Sciences, Tehran, Iran; 3. Cellular and Molecular Research Center, Urmia University of Medical Sciences, Urmia, Iran; 4. Dept. of Medical Parasitology and Mycology, School of Health, Urmia University of Medical Sciences, Urmia, Iran; 5. Dept. of Medical Entomology, School of Health, Urmia University of Medical Sciences, Urmia, Iran

**Keywords:** *Plasmodium berghei*, *Anopheles stephensi*, BALB/c, Malaria, Lifecycle

## Abstract

**Background:**

*Plasmodium berghei* is a rodent malaria parasite and has been very valuable means in the progress of our understanding of the essential molecular and cellular biology of the malaria parasites. Availability of hosts such as mice and vectors such as *Anopheles stephensi* has made this parasite a suitable system to study the parasite-host and vector-parasite relationships.

**Methods:**

This study was performed at Medical Entomology and Parasitology laboratories of the School of Public Health, Tehran University of Medical Sciences, Iran in 2016. The investigation was carried out to describe life cycle and parameters influencing maintenance of the parasite within the mice or the mosquito.

**Results:**

Results have revealed details and addressed some parameters and points influence maintenance of various life stages of the parasite including merozoites, macrogametocytes, ookinetes, oocysts and sporozoites in the laboratory model *P. berghei–A. stephensi*-BALB/c mouse. Injection of fresh infected blood results in higher gametocytemia in the animals. The more injected parasites result in earlier and higher parasitemia and exfelagellation centers in the mice blood. However, the highest number of infected mosquitoes and oocysts formation were observed when the parasitemia and exflagellation centers per microscopic field were 9% and 3.6 in the infected mice respectively. The infected mosquitoes should be maintained on 8% (w/v) fructose, 0.05% (w/v) PABA at 20±1 °C and 50%–80% relative humidity.

**Conclusion:**

This study helps to understand the biology of vertebrate-parasite and mosquito-malaria interactions that may aid in the development of a new generation of drug/vaccine and vector-based measures for malaria control.

## Introduction

Malaria remains one of the most prevalent tropical and infectious diseases in the world, with an estimated more than 200 million clinical cases every year (1). Attempts to restrict disease transmission have focused on using imagicides and adulticides to kill the larvae and adult mosquito vectors accordingly, the development of drugs to treat infected individuals, and hindering vector-human contact using physical barriers and repellents. The *Plasmodium* life cycle requires two different organisms to survive and develop: the vertebrate host and the female of *Anopheles* mosquitoes (1, 2).

In recent years, transmission-blocking strategies (TBS) are considered as a potential target for malaria control focused on mosquito stages of malaria parasites. The three main TBS included gametocytocidal drugs, transmission-blocking vaccines, and replacing wild mosquitoes with the ones harboring refractory traits. The first strategy is focused on exploring the gametocytocidal activity of commercially available anti-malarial drugs. The second TBS is based on immunological attack on sexual or mosquito stages of the parasite life cycle using different antigens including surface proteins of gametocytes and gametes, zygotes and ookinetes, as well as, antigens in the mosquito midgut surface such as calreticulin and alanyl aminopeptidase (AnAPN1), and other molecules such as chitinase (3–9). Some of these vaccine candidates are currently in preclinical phase and being considered for the first trials in humans (10). The third TBS is founded on changing the mosquito population towards a *Plasmodium*-resistant vector phenotype using an effector molecule triggering malaria refractoriness. Some of effector molecules were shown promising including phospholipase A2 (PLA2) to inhibit ookinete invasion and SM1 thought to block recognition sites for sporozoites and ookinetes (11, 12). Introduction of effector molecules can be achieved by releasing genetically modified (GM) mosquitoes (13), naturally refractory mosquitoes (11), artificial gene drive mechanisms (14, 15) and third-party modified organisms (paratransgenesis). The third party could be a symbiotic bacterium or fungus (16–20), and even viruses (21), modified to express effector molecule/s inside the mosquitoes. The midgut microbiome of some *Anopheles* is characterized and could be used as paratransgenesis means (22–27).

To assess the effectiveness of potential TBS that are able to disrupt the life cycle of the parasite in the mosquito vector, most studies have focused on *P. falciparum* and *P. berghei* and on the African mosquito, *A. gambiae*, and the Asian mosquito, *A. stephensi.* However*, P. berghei-A. stephensi* system is widely used when studying mosquito-*Plasmodium* interactions since *P. berghei* is a rodent malaria species, safe, unable of infecting humans, its manageability and exceptional robustness (28, 29). Numerous studies have described establishment of *P. berghei* life cycle and the rate of successful development from one life stage to the next within the mosquito and vertebrate hosts.

Since every developmental transition of the parasite exhibits strong level of success, here we tried to reveal essential requirements and optimized situation for a *P. berghei-A. stephensi-*BALB/c mouse laboratory model. Understanding the impact of variables influencing on life stage of the parasite is important to compare between potential TBS and their evaluations.

## Materials and Methods

### The host and vector

All gametocytes were raised in BALB/c mice and transmitted to *A. stephensi* strain Beech, where maintained at 20±1 °C and 50%–80% RH and fed on 8% fructose/0.05% para-aminobenzoic acid (PABA) (28). The mice were used to feed 24 h starved 3–5 d-old *A. stephensi* females for 30 min. In these experiments, more than 50 fed mosquitoes were assayed per sample. 24-h after feeding, unfed mosquitoes were removed by aspirator. Mosquitoes were then maintained on fructose [8% (w/v) fructose, 0.05% (w/v) PABA] at 20±1 °C and 50%–80% relative humidity. Day 9–10 post-feeding, mid-guts of a subset of lived specimens were dissected in a drop 0.5% mercurochrome and their midguts examined for oocysts by light microscopy. The remainder of each mosquito batch was incubated a further 8–10 d before counting the salivary gland sporozoites.

### The parasite

We followed the protocols previously described (30). To avoid the effect of parasite genetic variability, we used clones of the rodent malarial parasite *P. berghei* clone ANKA 2.34, a gift from Prof. Marcelo Jacob-Lorena of Johns Hopkins Bloomberg School of Public Health, Department of Molecular Microbiology and Immunology, Malaria Research Institute, Baltimore, USA.

All procedures were performed in accordance with the terms of the Iran Animals (Scientific Procedures) Act Project License and were approved by the Tehran University of Medical Sciences Ethical Review Committee (No. 26231).

The parasites were maintained in 4–10 wk old female BALB/c mice by serial mechanical passages (up to a maximum of 8 passages). To keep gametocyte infectivity to the mosquitoes, hyper-reticulocytosis was induced 2–3 d before infection by treating mice with 100 μL intraperitoneal (i.p.) phenylhydrazinium chloride (PH; 10 mg/mL in PBS) per 10 g mouse. Mice infections were monitored on Giemsa-stained tail-blood smears. Infections in mice were done on d 2–5 when a low but rising gametocytaemia succeeded (31).

## Results

We only addressed the hints and fine details normally not indicated exclusively in literature.

### Impact of anesthetic materials on BALB/c

The impact of kind of anesthetized materials on development of the parasite within mice, we tested two chemicals: Acepromazine and mix of Rompun® (Xylazine) and Vetalar® (Ketamin) (1:Xylazine/2: Ketamin/3: PBS). We injected intraperitoneal (i.p.) 50 μl (per mouse 15 gr) of the chemicals which anesthetizes the animal for 30–45 min. Acepromazine caused considerable reduction in the animal body temperature. This hindered the parasite development within the animal and also the cold animal was not attractive for mosquitoes to take blood meal.

### Hyper-reticulocytosis in BALB/c mice

Hyper-reticulocytosis was induced 2–3 d before infection by treating the mice with 100 μl (10 mg/mL in PBS) intraperitoneal (i.p.) phenylhydrazinium chloride (PH). Treating the BALB/c mice with 100 μL intraperitoneal (i.p.) phenylhydrazinium chloride (PH; 10 mg/mL in PBS) per 10 g animal weight was enough to induce hyper-reticulocytosis in the animals.

### Parasite storage

To store parasite-infected blood for further experiments it is recommended to use cardiac puncture method to harvest blood samples from infected mice. The blood should be collected into heparinized tubes and was immediately mixed with equal volume of PBS (30% glycerin), transferred to freezer (−20 °C) for 30 min, and then stored in −196 °C. It is possible to harvest 1.2 -1.3 and up to 1.7 ml blood from a BALB/c mouse weight 15–25 g.

### Injection of fresh/frozen infectious blood

**Fig. 1:**
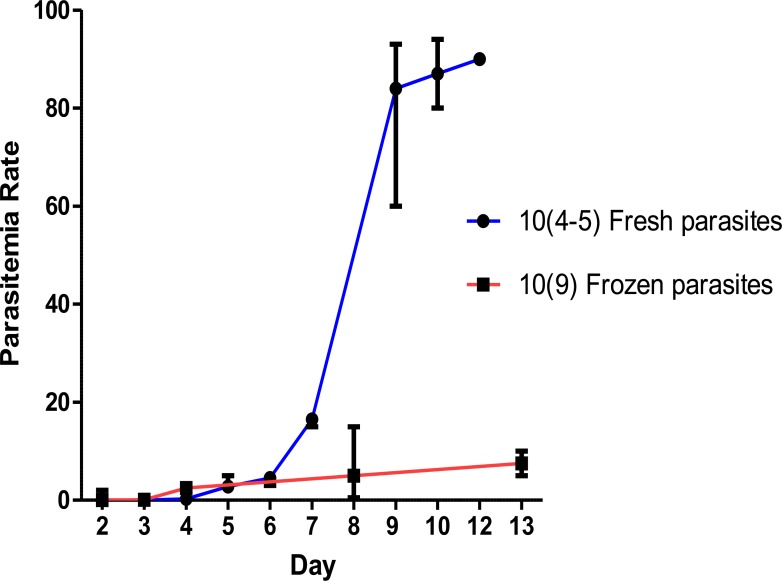
Temporal and level of *P. berghei* (ANKA clone 2.34) parasitemia in two groups of BALB/c mice received either 10^9^ parasites per ml frozen infectious blood or 10^4–5^ parasites per ml fresh infectious blood. Bars indicate median and error

The frozen infectious blood is used to infect noninfected mouse by injection of 200–300 uli.p. infected blood to a new mouse (20–25 g). It is recommended to thaw the frozen blood, incubate at room temperature up to 37 °C for a few minutes prior to injection. To make proper infection using infected frozen blood, it is recommended to inject the blood as soon as possible without treating the mice with PH. Appropriate parasitemia will appear in the mice several days post frozen-blood injection that is much later than the ones received fresh infected blood (Fig. 1).

**Fig. 2:**
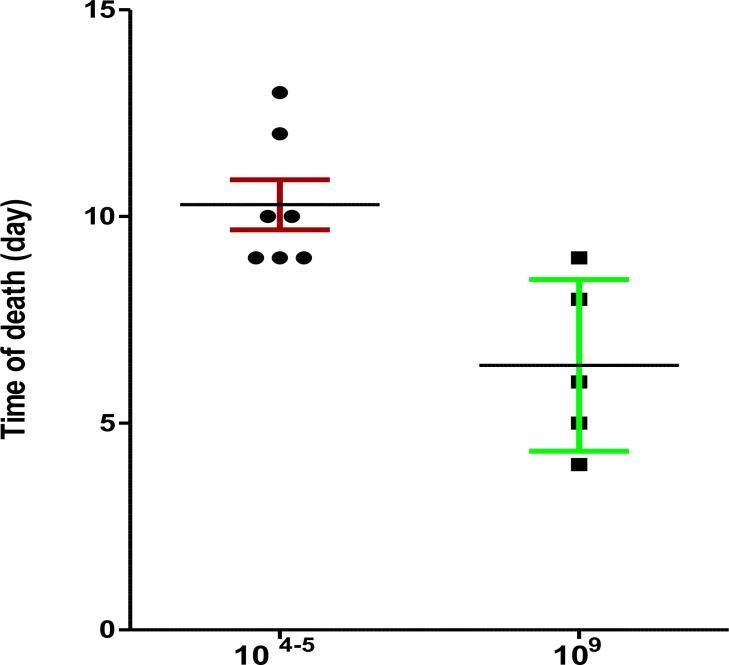
Time of death in two groups of BALB/c mice received either 10^9^ or 10^4–5^ *P. berghei* (ANKA clone 2.34) p/ml fresh infectious blood. Bars show means and SDs

The number of parasites in the fresh blood was 10^4^ to 10^5^ times less than the frozen blood. Injection of fresh infected blood with more than 50% parasitemia results in high gametocytemia in the animals but may cause early death in them. In two trails, one out of five (20%) and three out of ten (30%) mice died three days post-injection. In an independent experiment, the mean death time for the mice received 10^9^ parasites/ml (p/ml) infectious blood occurred at day 6 whereas the mean death time occurred at day 10 (4 d later) for the one received 10^4–5^ p/ml infectious blood (Fig. 2).

### Infectious symptoms in the mice

**Fig. 3:**
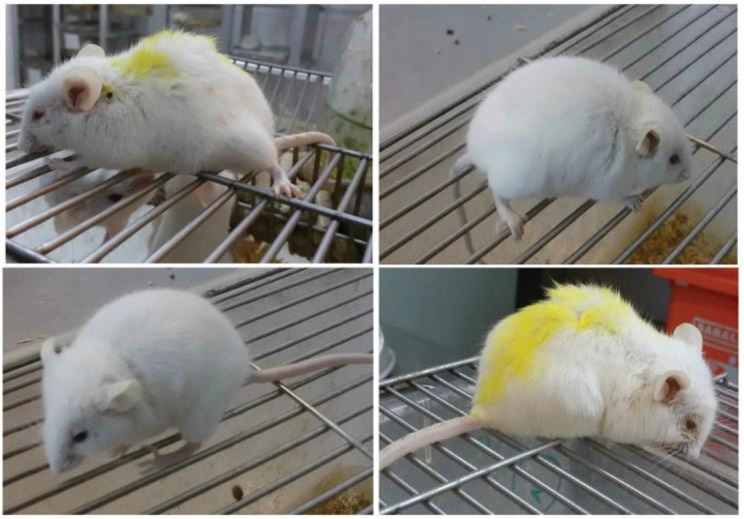
The symptoms of BALB/c mice infected by *P. berghei* ANKA clone 2.34

The symptoms of infected mice included secluding, temperature dysfunction, intense and fast breathing and increased heart rate, humping, blistering body hair (ruffled fur) particularly on back, eye weeping or closing, inactivation and reluctance to move in high parasitemia, hard walking, losing skin, ear discoloration, anemia and lightening the blood color from dark red to light red/fawn, RBC reduction, cerebral complications and death (Fig. 3).

### Obtaining P0 infected BALB/c mice

To obtain P0 infected mouse, the following hints are useful: 1) Injection of PABA into the non-infected mice 3 d prior to injection of infected blood, 2) parasitemia in the donor infected mouse should be more than 40%, 3) injection of 230–250 ul of infectious blood to non-infected BALB/c mouse is enough, 4) prior to injection, the mouse should be maintained in 23±2 °C, 12:12 dark: light photoperiod regime, 5) two to three days post injection is the best time for counting exflagellation centers.

### Effect of number of parasites on parasitemia and exfelagellation centers

**Fig. 4:**
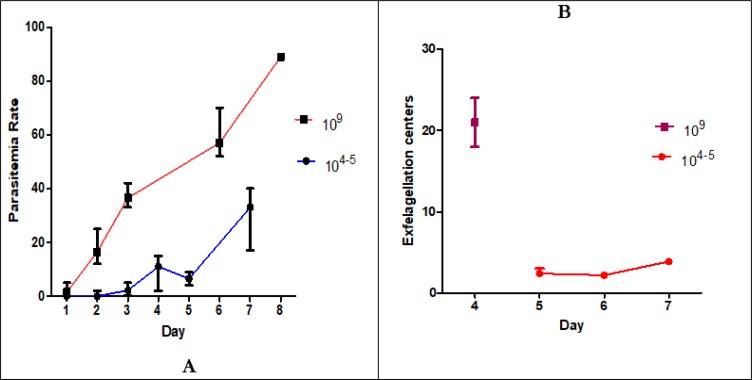
Effect of number of injected *P. berghei* (ANKA clone 2.34) parasites on the temporal and level of parasitemia (A) and exfelagellation centers (B) in BALB/c mice. Bars indicate medians and errors

There is positive correlation between number of injected parasites and level and or temporal parasitemia in the BALB/c mice (Fig. 4). The more injected parasites result in earlier and higher parasitemia and exfelagellation centers in the mice blood.

### Oocyst formation in A. stephensi mosquitoes

**Fig. 5:**
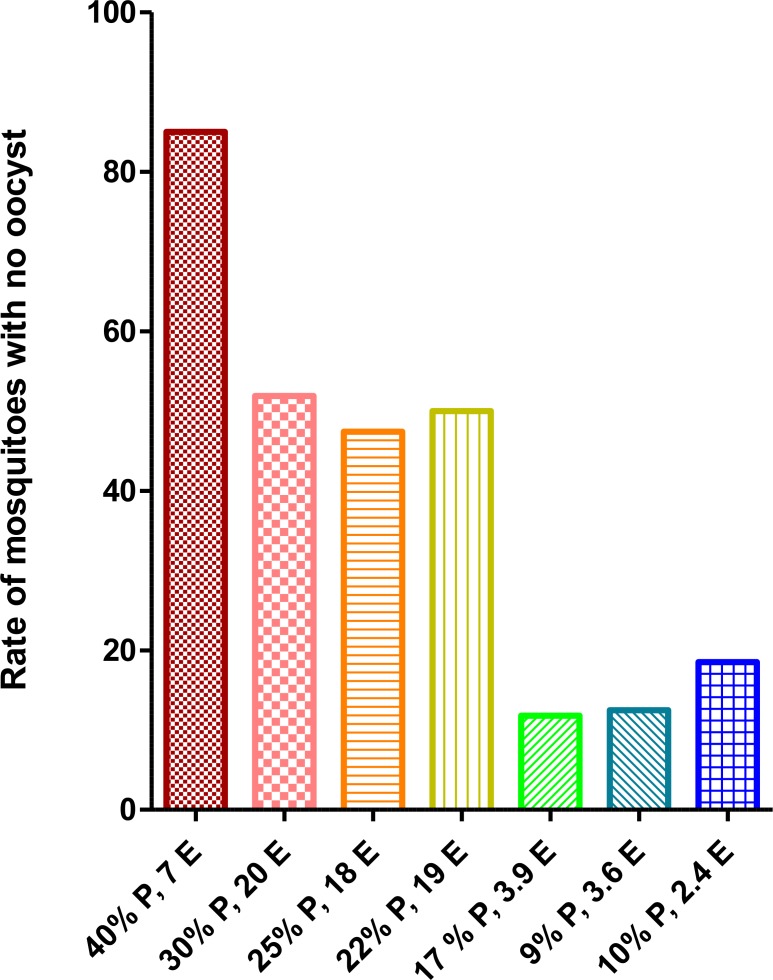
Relationship between rate of parasitemia (P)/number of exflagellation centers (E) of *P. berghei* (ANKA clone 2.34) per microscopic field in BALB/c blood and frequency of *A. stephensi* mosquito midgut with no oocyst

Feeding the mosquitoes on the infectious mice with high parasitemia or exflagellation centers in each microscopic field result in high oocyst formation, however, the number of mosquitoes with no oocyst increased dramatically (>47–85%) (Fig. 5).

**Fig. 6:**
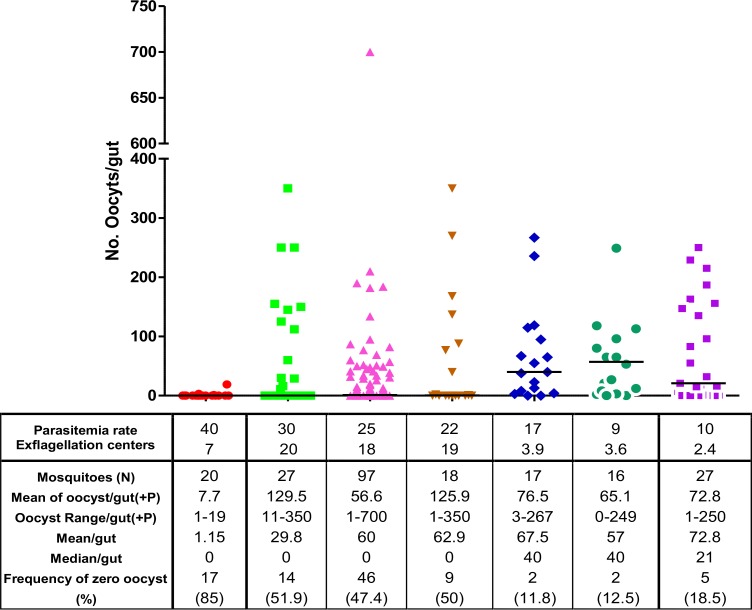
Effect of parasitemia/number of exflagellation centers per microscopic field of *P.berghei* (ANKA clone 2.34) on oocyst formation in *A.stephensi* midgut. Bars show median. +p: mosquitoes with at least one oocyst

High [7–20] exflagellation centers per microscopic field or high parasitemia (>17%) intensify number of mosquitoes with no oocyst. On the other hand, using infectious mice with 2.4–3.9 exflagellation centers in each microscopic field and 9%–17% parasitemia results in high frequency of infected mosquitoes and oocysts formation (Fig. 6). The best results were observed when the parasitemia and exflagellation centers per microscopic field was 9% and 3.6 in the infected mice respectively (Fig. 6).

The best time for feeding mosquitoes on infected mouse is when 3-4 exflagellation centers exist in each microscopic field. Based on the number of parasites injected into the mouse, this situation happens throughout days 3–4 and 5–6 post-injection when respectively 10^9^ and 10^4–5^parasites injected (Fig. 3).

Overall, 4-7 d old mosquitoes should be maintained on 8% (w/v) fructose, 0.05% (w/v) PABA at 20±1 °C and 50%–80% relative humidity from the time of adult emergence. The female mosquitoes were kept starved for 12 h before blood meal. The hunger females should be separated and transferred to a new cage. The infected mouse was anesthetized by Ketamin/Xylasine and put on top of the cage where the room temperature was about 20±1 °C.

It is highly recommended to warm the animal body by putting a warm cotton pad on its body and changing the pad every 5 min. This helps to keep the animal body warm enough and nullify cooling effect of the anesthetic compounds. Totally, 20–30 min is enough for most (>90%) of females to take blood meal. The fed mosquitoes should be maintained on 8% (w/v) fructose, 0.05% (w/v) PABA at 20±1 °C and 50%–80% relative humidity through the experiments. The fructose-PABA vial should be changed every two days. The vial stocks should be kept at 20 °C before use. Usually, about 60%–80% of fed females will be alive 15–20 d post blood meal at optimized condition. The mosquitoes lay eggs 4–5 d post blood meal at 20±1 °C.

The parasite zygotes and ookinetes could be seen accordingly 1 and 24 h after infected blood intake. To support survival rate of the female mosquitoes, it is recommended to offer two more blood meals (from a non-infected mouse) on day six and 12, following laying eggs by the females. Day 9–10 and 20 post blood meal is the best time for oocyst and sporozoite observation accordingly. Oocysts can be counted by phase contrast microscopy of freshly dissected midguts. Sporozoites can be similarly counted on days 20 onwards by hemocytometry of dissected salivary glands of alive mosquitoes immediately prior to dissection.

**Fig. 7:**
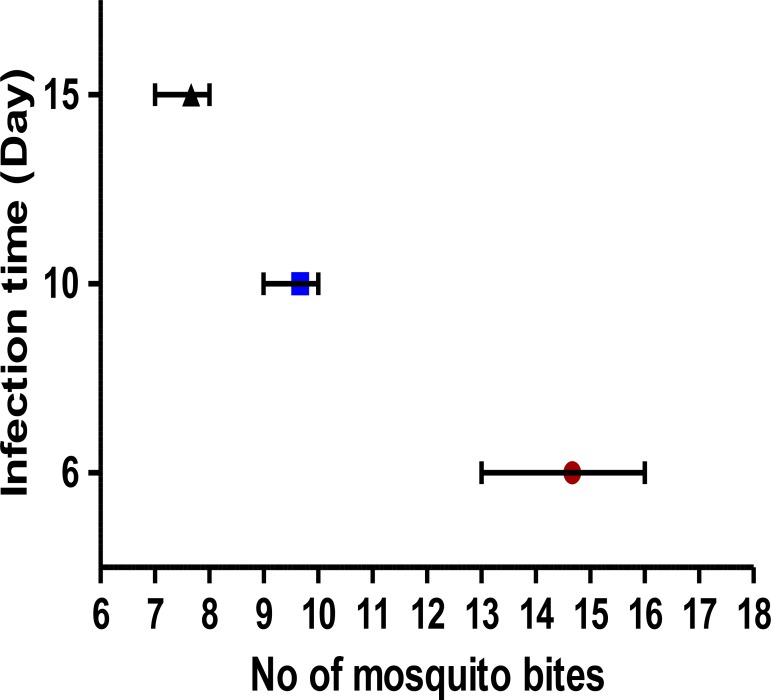
Relationship between numbers of *P. berghei* infected *A. stephensi* bites and infection time in the BALB/c blood. Prevalence (number of mosquitoes with at least one oocyst) of infected mosquitoes was 80%. The bars indicate means with ranges

If the mosquitoes had sporozoites they are ready for parasite transmission to a new host. To ensure transferring the parasites from infected mosquitoes to a new mouse, although theoretically one mosquito is enough, it is recommended to use at least 10 infected females per mouse because all of the mosquitoes may not be infected and one mosquito may not able to inoculate enough parasite to the host. Younger mice with 10–15 g weight are preferred to older ones with more weight for parasite transmission. In our experiments, the P0 parasites were observed on day six post mosquito bites in the mice (n=3) who received 13–16 mosquito bites. Other mice group (n=3) received fewer bites (7–8 and 9–10 bites) the parasites appeared on average day 15 and 10 post mosquito bites accordingly (Fig. 7). Further analysis showed that rate of sporozoite infection in the mosquitoes used in this experiment was 80%.

Following mosquito bites, the sporozoites enter the liver cells (hepatocytes) less than an hour (30 min), and then after 48 h, the merozoites form are released into the bloodstream to attack red blood cells (RBC). In RBC, they replicate asexually in 22–24-h cycle. When parasitemia of P0 reach to 20% in the mice blood, the blood could be collected (10 ul blood + 90 ul PBS/mouse 15g) and injected to new mice to prepare P1 parasites. It takes almost one week until the parasites (P1) appears in the mice. When the parasitemia (P1) reach 30%–50%, the blood can be taken and stored in freezer for further investigation. For preparing P2 parasites, the mouse blood containing P1 parasites with 20%–30% parasitemia could be injected into new mice. The new host (mouse) should be treated with PH three days in advance of receiving the parasites. The P2 parasites will appear in the mice 3–4 d later. The mice with P2–P7 parasites are suitable for mosquito feeding for TBS assays.

## Discussion

We have revealed fine details for maintaining *P. berghei-A. stephensi*-BALB/c mouse system which is crucial and very helpful for studies involving malaria transmission blocking assays. Although the system we explained here was established for *P. berghei-A. stephensi*, it has the potential applicability to other malaria vectors. *P. berghei* is able to complete its life cycle in different mosquito species (30, 32, 33). However, when studying mosquito-malaria interactions, one should consider the vector intrinsic factors comprising *Anopheles* species gametocyte activating factor(s), pH, temperature, and quality and quantity of blood ingestion which may influence on the mosquito infectivity outcome (34, 35).

We used BALB/c mice as a vertebrate host for *P. berghei*, however, other rodents including Swiss Webster mice, Theiler’s Original (TO–outbred), and C57BL/6 have been used for similar systems. The quality and quantity of inhibitory factors present in the infected blood of different mice such as antibodies, metabolites, and cytokines (36, 37) may enhance or diminish the mosquito infectivity outcome. Further investigation needs to clarify the effect of the parasite/host combinations on the maintenance of the parasite in vivo (38).

For the lab with less security level, this system present here is preferred to the systems use *P. falciparum* since *P. berghei* is a rodent malaria species and is safe and unable of infecting humans (29). However, the systems involved human malaria species such as *A.stephensi/A.gambiae–P. falciparum* might vary from the rodent system we present here particularly in the gametocytemia and the mosquito infectivity.

In our experiments, the number of infectious parasites ingested by the mosquito from the mouse has great effect on the success of *Plasmodium* infection in the mosquitoes. Although the number oocystes increased in the mosquitoes fed on high parasitemic mouse, number of infectious mosquitoes reduced intensely which is in accordance with the finding of other researchers (35, 39–44). Therefore it is highly recommended for researchers who use *P. berghei*-systems to use infected mouse or its infected blood with low gametocyte densities (2–3 exflagellation centers per microscopic field) when they evaluate TBS and calculate value of interventions. Here we showed that more inoculated sporozoites accelerate the parasitemia in the host. In addition to the number of mosquito bites, the number of inoculated sporozoites and the host-related factors may affect the quality and quantity of parasitemia in the host. This is in agreement with previous studies indicating the positive relationship between parasitemia and number of infected mosquito bites or sporozoites (31, 45, 46).

The results of this study showed that between the two parameters of parasitemia and exflagellation centers, number of exflagellation centers has more influence on the rate of infected mosquitoes and proper number of oocyst formation. However, this depends on mosquito strain, amount of blood intake by mosquito, number of active gametocytes in the blood, and some other unknown factors in the blood (8, 47).

## Conclusion

The results of this study showed that various factors influence on the maintenance of *P. berghei-A. stephensi*-BALB/c mouse system. This information helps to understand the biology of vertebrate-parasite and mosquito-malaria interactions that may aid in the development of a new generation of drug/vaccine and vector-based measures for malaria control.
